# Chemical composition and pharmacological activities of *Pisum sativum*

**DOI:** 10.1186/s12906-017-1699-y

**Published:** 2017-03-27

**Authors:** Md Nazmul Hasan Zilani, Tamanna Sultana, S. M. Asabur Rahman, Md Anisuzzman, Md Amirul Islam, Jamil A. Shilpi, Md Golam Hossain

**Affiliations:** Pharmacy Discipline, Life Science School, Khulna University, Khulna, 9208 Bangladesh

**Keywords:** HPLC, LCMS, Ellagic acid, β-sitosterol, Antioxidant, Antidiabetic

## Abstract

**Background:**

Consumption of vegetables has been proven to be effective in the prevention of different diseases. Traditionally edible aerial part of *Pisum sativum* L. subsp*. sativum* (Fabaceae) is used to treat diabetes, heart diseases and as blood purifier. Present study was aimed to explore the traditional use of aerial parts of *P. sativum* as a source of antidiabetic agent. In addition, antioxidant activity and chemical composition was carried out.

**Methods:**

Total polyphenol content was spectrophotometrically determined using Folin Chiocalteu’s reagent while the flavonoids by aluminum chloride colorimetric assay. Identification of compounds of the extract was made through HPLC and LCMS. Antihyperglycemic activity was assessed by oral glucose tolerance test in mice. Antioxidant activity was determined by DPPH free radical scavenging and reducing power assay.

**Results:**

Total polyphenol and total flavonoids content were found to be 51.23 mg gallic acid equivalent and 30.88 mg quercetin equivalent per gram of dried plant extract respectively. Ellagic acid and *p*-coumeric acid were detected through HPLC. A total of eight compounds including naringenin, β-sitosterol were indentified through LCMS. In OGTT, extract (200 mg/kg bw) showed a 30.24% decrease (*P*< 0.05) in blood glucose levels at 30 min compared to the normal control. The extract showed IC_50_ value of 158.52 μg/mL in DPPH scavenging assay and also showed comparable reducing power.

**Conclusion:**

Along with other compounds ellagic acid and β-sitosterol present in the extract may be responsible for its antioxidant as well as antihyperglycemic activities. Altogether these results rationalize the use of this vegetable in traditional medicine.

## Background

In hyperglycemia, intracellular carbohydrate metabolism is impaired followed by production of copious number of Reactive Oxygen Species (ROS) by various processes namely glucose oxidation, glucose toxicity and oxidative phosphorylation [1]. In this condition the most devastating ROS engendered in plasma are superoxide anion, hydroxyl and peroxynitrite radicals [2]. Glucose oxidation is believed to be the main source of these free radicals that are responsible for destruction of the pancreatic beta cells responsible for producing insulin [2]. Not only are these radicals involved in the cause of diabetes, they also appear to play a role in some of the complications seen in long-term treatment of diabetes. Hence, using antioxidants can be helpful for mopping up these reactive oxygen species. Traditionally natural sources have been used to treat diabetes for a long period of time [3]. About 400 traditional plants including edible herbs have been used to treat diabetes [4]. Antidiabetic activity of edible herb *Allium cepa, Allium sativum, Dioscorea dumetorum, Momordica charantia, Coccinia indica, Momordica cymbalaria* has been reported [4, 5]. Edible herbs contain significant bioactive nutritional and health-promoting ingredients with antidiabetic and antioxidant activities [6]. So, herbal plants may be an alternative way of prevention or treatment of diabetes and other fatal diseases caused by reactive species.

*P. sativum* L. (Fabaceae), locally known as matar, is an annual or perennial herb. It is cultivated throughout the Bangladesh [7]. Traditionally seeds are used as nutrient, appetizer, refrigerant, laxative, astringent and also used in treating wrinkled skin, diabetes, acne, phlegm and intestinal inflammation [7, 8]. Antioxidant, antimicrobial and hypoglycemic activities of seeds also have been reported [9]. Furthermore, pericarp of pods showed potential antihyperglycemic activity [10]. Fruits and seeds contain starch, albuminoids, alkaloids, galactolipids, trigonelline, piplartine and essential oils [11]. Petiole and tendril yielded kaempferol-3-triglucoside, quercetin-3-triglucoside, and their p-coumaric esters [12, 13]. Newly growth tender (leaves and stem) is used as vegetables in Bangladesh [14]. It is traditionally used in treatment of diabetes, heart diseases and as blood purifier [14].

Literatures reveals that there are very few studies have been reported regarding biological activity or chemical composition of the edible aerial parts of *P. sativum.* As a part of the continuation of our research on bioactivity screening of Bangladeshi medicinal edible herbs, present investigation was carried out to evaluate the usefulness of the *P. sativum* extract in diabetes by oral glucose tolerance test. It was also tested for antioxidant activity and defined chemically by HPLC and LCMS analysis.

## Methods

### Chemicals and reagents

Gallic acid, caffeic acid, *p*-coumaric acid, (+)-catechin hydrate, (−)-epicatechin, vanillic acid, rutin hydrate, ellagicacid, kaempferol, myricetin, quercetin, 2, 2-Diphenyl-1-picryldydrazyl (DPPH^.^), ascorbic acid and butylated hydroxy toluene were purchased from Sigma–Aldrich (St. Louis, MO, USA). HPLC grade methanol, acetonitrile, acetic acid and ethanol were obtained from Merck (Darmstadt, Germany). Standard drug Glibenclamide hydrochloride was purchased from Square Pharmaceuticals Ltd., Bangladesh.

### Plant materials and extraction

The edible aerial part of *P. sativum* L. was collected in December 2014 from Khulna, Bangladesh and identified by experts at Bangladesh National Herbarium, Dhaka, Bangladesh. A voucher specimen (DACB 41155) has been submitted there for future reference. The shade dried and grinded powder (500 g) was soaked in 98% ethanol. After removal of debris and evaporation of solvent by rotary evaporator crude extract (yield 2.8% *w*/*w*) was found and stored at 4 °C until experiment commenced.

### Experimental animals

In the present study Swiss-albino mice of average weight 22-25 g and 4-5 weeks age were used. Mice were housed in an ambient room temperature of 24 ± 1 °C; 12 h light and dark cycle with controlled humidity. Mice were allowed standard pellet diet and water ad libitum. Ethical guidelines of Organization for Economic Cooperation and Developmentwere followed to carry out the experiment.

### Identification of Phytochemical constituents

To identify the presence of therapeutically active phytochemicals namely flavonoids, polypheols, alkaloids, glycosides, terpenoids, and saponins, standard chemical tests were carried out [15].

### Total polyphenol content

To determine the total polyphenol content of the extract, extract solution (1 mg/ml) was mixed with 5 mL of ten times diluted Folin-Ciocalteu reagent. In that mixture, 4 mL of 75 g/L sodium carbonate was added. After incubation period of 30 min at 40°C absorbance of the reaction mixture was measured at 765 nm using Shimadzu UV visible spectrophotometer (Model 1800, Japan). Gallic acid (0-1 mg/mL) was used to prepare standard calibration curve. Gallic Acid Equivalent (GAE) was determined (mg/mL) from the equation of calibration line and then total polyphenol content was expressed in terms of mg of gallic acid equivalent per gram of dry extract [16].

### Total flavonoids content

A well-known aluminum chloride colorimetric method was used to determine the total flavonoids content of the extract [17]. In the extract solution(1 mg/ml), 4 mL distilled water and 0.3 mL of 5% *w*/*v* sodium nitrate was sequentially mixed. Five minutes later, 0.3 mL of 10% *w*/*v* aluminum chloride was added to the mixture with continuous shaking. At the sixth minute, 2 mL of 1 M sodium hydroxide was added and the volume was adjusted to 10 mL. Then absorbance was measured at 510 nm. For this assay quercetin (0-1 mg/mL) was used for standard calibration curve. After reading the quercetin equivalent from the calibration line, total content of flavonoids was expressed as mg quercetin equivalent (QE) per gram of dry plant extract.

### HPLC detection of polyphenol

To detect the presence of polyphenols present in the extract, HPLC analysis was carried out on Rapid Separation LC (RSLC) systems (Thermo Fisher Scientific Inc., MA, USA), coupled to separation pump (LPG-3400RS), Ultimate 3000RS autosamplier (WPS-3000) and diode array detector (DAD-3000RS). Separation was carried out at 30 °C using Acclaim® C18 (4.6 × 250 mm; 5 μm) column (Dionix, USA) and injection volume of 20μl. Gradient method was applied as 5%A/95%B, 0 min; 10%A/80%B/10%C, 10 min; 20%A/60%B/20%C, 20 min and 100%A at 30min and 5min post run with solvent A. For spectrophotometric detection, the wavelength was optimized at 280 nm for first eighteen min, 320 nm for next six minand finally to 380 nm for the rest of the analysis and the diode array detector was set at a range from 200 to 70 nm. Acetonitrile (solvent A), acetic acid solution pH 3.0 (solvent B), and methanol (solvent C) were used as mobile phase with flow rate at 1 mL/min.

For the calibration curve, gallic acid, (+)-catechin hydrate, vanillic acid, (−)-epicatechin, *p*-coumaric acid, ellagic acid, myricetin, kaempferol (1.0-5.0 μg/mL);(+)-catechin hydrate, caffeic acid, rutin hydrate (0.5-4.0 μg/mL) and quercetin (0.25-3.0 μg/mL) were used as standard. Extracts solution (5.0 mg/mL) were prepared in ethanol by vortex mixing for 30 min. Prior to HPLC analysis mixed standards, sample and spiked solutions were filtered through 0.20 μm nylon syringe filter and degassed in an ultrasonic bath for 15min [18].

### Chemical profiling through LCMS

Liquid Chromatography Mass Spectroscopy (LCMS) analysis was performed using Agilent 6530 Accurate-Mass Q-TOF LC-MS system. It equipped with a reversed-phase C18 analytical column of 50 mm × 2.1 mm × 1.8 μm particle size (Agilent 6530). The column oven temperature was set at 35 °C, and the flow rate was 250 μL/min. Mobile phases A and B were water and acetonitrile, respectively, each containing 5 mM ammonium formate and 0.1% formic acid. The linear gradient programme was set as follows: 0 min, 100% A; 45 min, 100% B; 50 min, 100% B; 55 min, 100% A. The injection volume was 20 μL with a run time of 15 min. The UHPLC was hyphenated to a triple quadrupole mass spectrometer 3200 QTrap (ABSciex) equipped with an electrospray ionization interface set at negative mode. The interface heater held at the temperature of 500 °C and an ion-spray (IS) voltage of −4500 eV. The nebulising gas (GS1), heating gas (GS2) and curtain gas pressures set at 40, 40 and 10 psi, respectively during the whole analysis. Nitrogen was used as collision and spray gas. Full scan data acquisition was performed, scanning from m/z 5 to 1500 in enhanced MS IDA EPI mode [19].

### Oral glucose tolerance test

Oral glucose tolerance was assessed using overnight (16 h) fasted mice. It was divided into four groups containing five mice in each group. Group I was treated as control and group II received standard glibenclamide (5 mg/kg, orally). Group III and IV were orally treated with extract of 100 and 200 mg/kg body weight respectively. Glucose (3 g/kg) was administered orally after 30 min of oral administration of extract. Blood samples were collected from tail vein at 0, 30, 60, 90 and 120 mins after glucose load. The blood glucose levels were analyzed using glucose test strips and glucose meter (EZ Smart-168, Tyson Bioresearch Inc., Taiwan) [17].

### Acute toxicity test

Acute oral toxicity of the extract was assessed in mice according to the guidelines of the Organization for Economic Cooperation and Development [17]. The mice were fasted overnight (16 h), divided into 5 groups (*n* = 5) and the extract was orally administered at the dose of 100, 200,400 and 800 mg/kg body weight. The control group received distilled water. Individual observations for lethality and any physical sign of toxicity of mice were started during the first two hours continuously and then at six hours interval for 24 h time period and finally after every 24 h up to 14 days.

### DPPH free radical scavenging assay

A stock solution of 1024 μg/mLwas prepared to determine the DPPH radical scavenging activity of the extract. Different concentrations (512, 256, 128, 64, 32, 16, 8, 4, 2, 1 μg/mL) were obtained through serial dilution of stock solution. In 3mL of freshly prepared 0.004% *w*/*v* DPPH solution, 1mL sample solution was added. After incubation of 30 min at dark place, absorbance of each concentration was measured at 517 nm. Ascorbic acid was used as standard free radical scavenger. The scavenging activity of the samples was calculated using the formula: % scavenging activity = [(Abs_c_-Abs_s_)/Abs_c_] × 100; where Abs_c_ is the absorbance of control and Abs_s_ is the absorbance of extract or standard. Concentration required to scavenge 50% of free DPPH (IC_50_ value) was estimated from the obtained data [18].

### Reducing power assay

To determine the reducing power of the extract, from the stock solution different concentrations (0.1-1 mg/mL) of extract was prepared. In 1 mL of sample solution, 0.2 M phosphate buffer (2.5 mL; pH 6.6) and 10 g/L potassium ferricyanide (2.5 mL) were added. The mixture was incubated at 50°C for 20 min. After cooling at room temperature, 100 g/L trichloroacetic acid (2.5 mL) was added. The mixture was centrifuged at 3000 rpm. For 10 min. In the supernatant (2.5 mL), distilled water (2.5 mL) and 1 g/L ferric chloride (0.50 mL) were added. After 10mins, absorbance of the mixture was measured at 700 nm. Butylated Hydroxy Toluene (BHT) was used as standard [20].

### Statistical analyses

Data acquisition, peak integration and calibrations in HPLC were performed with DionixChromeleon software (Version 6.80 RS 10). Analyst software version 1.5.2 was used for method development, data acquisition and data processing in LCMS. Statistical significance of oral glucose tolerance test was estimated by student’s *t*-test. All values were expressed as mean ± SD of three parallel measurements.

## Results

**Fig. 1:**
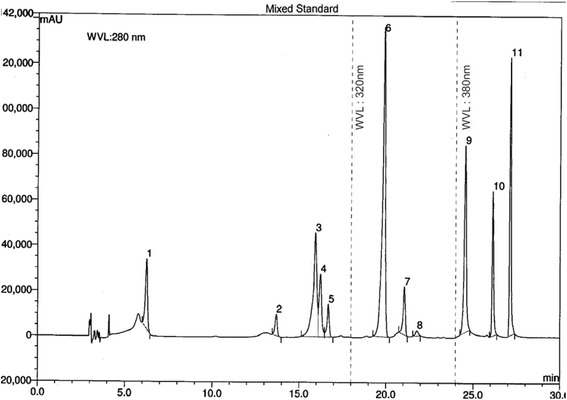
HPLC chromatogram of a standard mixture of polyphenolic compounds (peak 1: gallicacid, 2:(+)-catechin, 3:vanillic acid 4: caffeic acid 5: (−)-epicatechin 6: *p*-coumaric acid 7: rutin hydrate 8: ellagic acid 9: myricetin, 10: quercetin, 11: kaempferol)

**Fig. 2:**
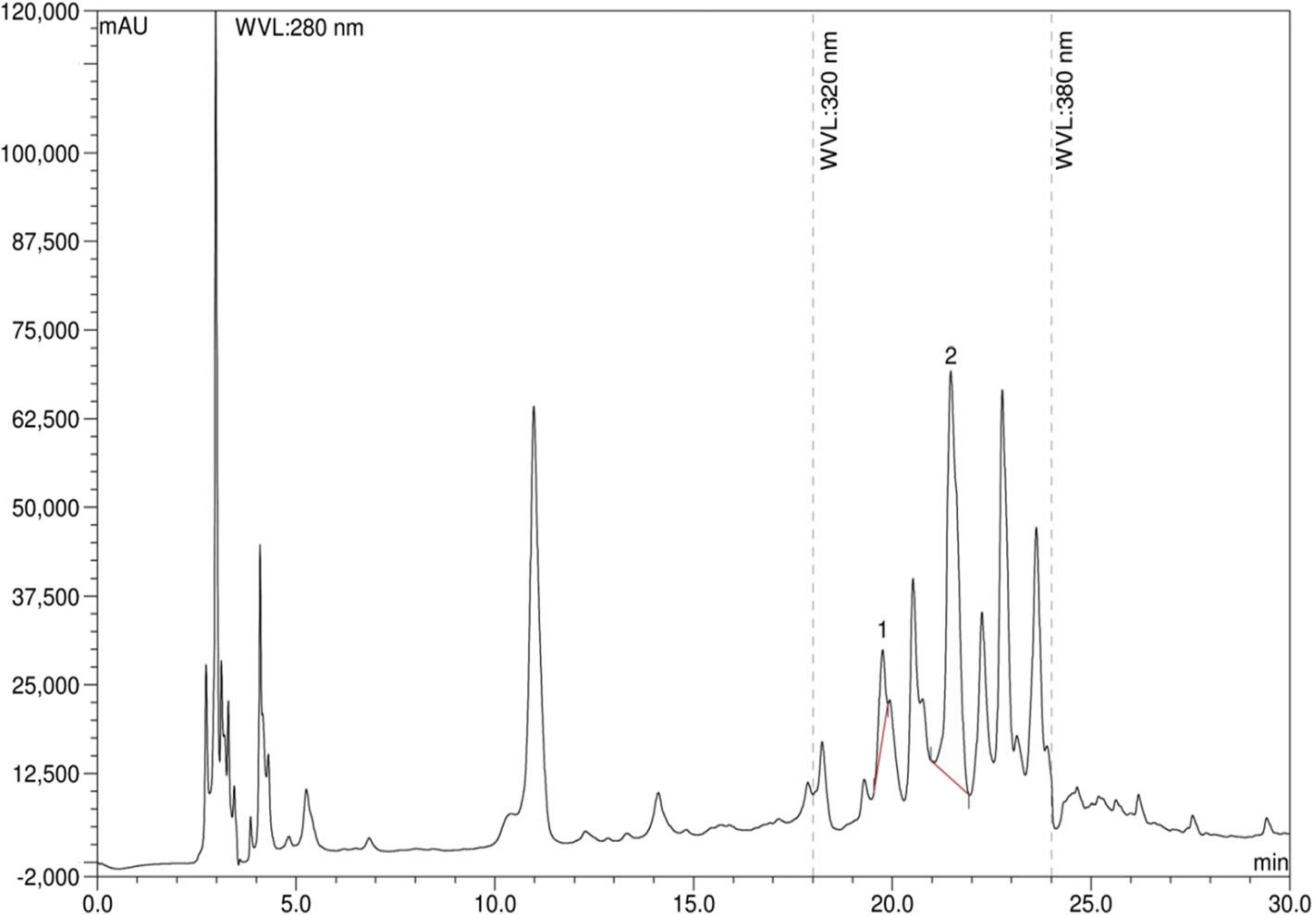
HPLC chromatogram of ethanol extract of *P. sativum* (peak 1: *p*-coumaric acid, 2: ellagic acid)

**Fig. 3:**
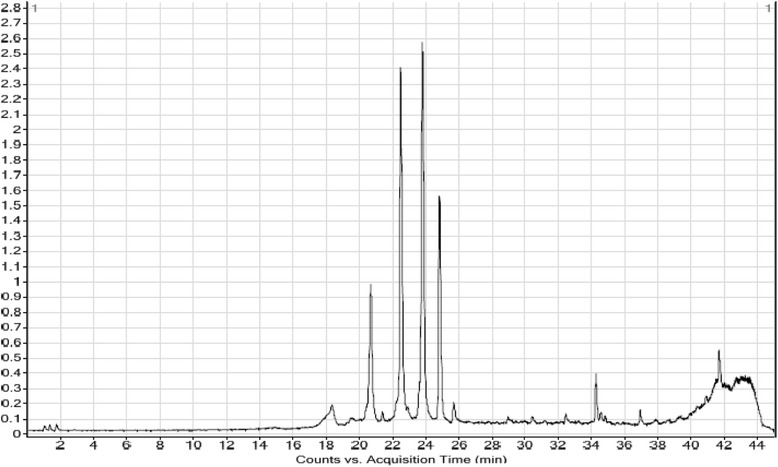
LCMS chromatogram of the extract

In the preliminary phytochemical screening, the extract showed the presence of therapeutically active phytochemicals flavonoids, polyphenols, alkaloids, glycosides, terpenoid and absence of saponins. To determine total phenol content acalibration curve was prepared from graph of concentration versus absorbance of standard gallic acid. The equation y = 8.043+ 0.213, R^2^ = 0.985 was obtained from that curve. Total phenolic content of the extract was calculated and found to be 51.23 mg gallic acid equivalent per gram of dry extract. Quercetin as standard was used to obtain a calibration curve with the equation y = 1.169+ 0.008, R^2^ = 0.994 from which total flavonoid content of the extract was calculated as 30.88 mg QEg^−1^ dry extract. In HPLC analysis of polyphenols, eleven standard polyphenol compounds were used for comparison. Among these compounds extractcontained a very high concentration of ellagic acid (899.19 mg per 100 g of dry weight) and fairly low (7.78 mg per 100 g of dry weight) amount of *p*-coumaric acid. The chromatographic profiles of standard polyphenol compounds and extract were presented in Figs. 1 and 2. The concentrations of present phenolic compounds were calculated based on the calibration curves of the standards and reported in Table 1. The LCMS base peak chromatogram of the ethanol extract was shown in Fig. 3. From LCMS data and the related literatures β-sitosterol, β-amyrin, kaempferol-3-neohesperidoside, 6-prenylpinocembrin, naringenin, kaempferol-3- glucoside, 1,2-benzenedicarboxylic acid, 6H-Benzofuro[3,2-c][1] benzopyran and diisooctyl esterwere identified in the extract. The chromatographic and LCMS data including retention time, experimental and calculated M + H, molecular formulas and proposed compounds were summarized in Table 2.

**Table 1:** Contents of polyphenolic compounds in *P. sativum* extract

Polyphenolic Compound	Content (mg/100 g of dry extract)	% RSD^a^
*p*-Coumaric acid	7.78	0.19
Ellagic acid	899.19	6.83

^a^ *RSD* Relative Standard Deviation

**Table 2:** LCMS data of the compounds identified in the extract

RT (min)	M + H experimental	Molecular formula	M + H calculated	Suggested compound	Reference
1.013	427.0791	C_30_H_50_O	426.386	β-amyrin	[42]
20.403	285.1232	C_17_H_16_O_4_	284.30	6H-Benzofuro[3,2-c][1]benzopyran	[43]
21.279	415.2408	C_29_H_50_O	414.71	Β-sitosterol	[44]
22.959	391.28	C_24_H_38_O_4_	390.55	1,2-Benzenedicarboxylic acid, diisooctyl ester	[45]
23.680	595.45	C_27_H_30_O_15_	594.51	kaempferol-3-neohesperidoside	[46]
34.316	325.22	C_20_H_20_O_4_	324.3704	6-prenylpinocembrin	[47]
36.622	273.18	C_15_H_12_O_5_	272.25278	Naringenin	[48]
40.302	449.3385	C_21_H_20_O_11_	448.38	Kaempferol-3- glucoside	[46]

**Fig. 4:**
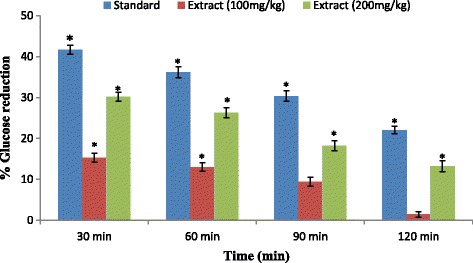
Percent reduction of blood sugar content by the different doses of extract

The antihyperglycemic effects of the *P. sativum* extract on the oral glucose tolerance test (OGTT) in nondiabetic micewere presented in Table 3. The maximum plasma glucosein the normal control group was at 30 min after glucose (3 g/kg) loading and then gradually declined. The treatment with different doses of extract (100 and 200 mg/kg) significantly (*P*< 0.05) reduced blood glucose levels by 18.15%, 30.24% at 30min respectively, and significant (*P*< 0.05) reduction in the following hours was observed only at 200 mg/kg treated mice (Table 3 and Fig. 4). Mice treated with oral antidiabetic reference drug glibenclamide showed significant (*P*< 0.05) reduction in blood glucose levels at 30, 60, 90, and 120 min after glucose load compared to normalcontrol (Table 3 and Fig. 4). The oral acute toxicity evaluation of the extract at 100, 200, 400 and 800 mg/kg body weight resulted in no mortality and no visible signs of acutetoxicity throughout the 14 days. These results indicated that the LD_50_(lethal dose in 50%) value of the extract was greater than 800 mg/kg body weight.

**Table 3:** Effect of extract on mice during oral glucose tolerance test

Treatment		Blood glucose level ^a^ (mmol/L)			
	0 min	30 min	60 min	90 min	120 min
Control (10 mL/kg)	5.28 ± 1.42	12.40 ± 3.16	10.92 ± 2.56	7.95 ± 0.59	6.8 ± 0.08
Glibenclamide (5 mg/kg)	5.06 ± 1.32	7.54 ± 3.62*	6.97 ± 2.63*	5.54 ± 1.28*	5.3 ± 0.95*
*Extract* (100 mg/kg)	5.16 ± 2.11	10.15 ± 2.76*	9.28 ± 3.12*	7.2 ± 2.63	6.7 ± 3.24
*Extract* (200 mg/kg)	4.94 ± 2.11	8.65 ± 3.28*	8.05 ± 2.54*	6.5 ± 2.29*	5.9 ± 2.34*

^a^ Values represent as mean ± SD, *n* = 5, ^*^ *P* < 0.05 vs. control, Student’s *t*-test

The DPPH radical scavenging activity of the extract (IC_50_ = 158.52 ± 5.46 μg/mL) was approximately ten fold lesser than that of the standard ascorbic acid (IC_50_ = 14.70 ± 3.24 μg/mL). A concentration dependent reducing capacity of the extract was found. At the concentrations of 0.1, 0.2, 0.3, 0.4, 0.5 and 1 mg/mL extract showed absorbance of 0.092, 0.123, 0.205, 0.216, 0.239, and 0.544 while standard butylated hydroxytoluene showed absorbance of 0.467, 0.733, 1.059, 1.107, 1.242, and 1.472, respectively.

## Discussion

Medicinal plants are traditionally used for therapeutic purposes. Secondary metabolites of plants are mainly responsible for therapeutic action in biological system [21]. Biological actions depend on the concentration of secondary metabolites in plant cells that fluctuate along with seasons, climates, particular growth phases, plant parts and extracting solvent [22]. Leaves are one of the highest sources of bioactive compounds [22]. Some studies have suggested that consumption of commonly used vegetables has been proven to reduce the risk of life threatening diseases as vegetables have appeared for noteworthy sources of bioactive compounds [23].

Oxidative stress is one of the major causes of reduced glucose tolerance [24]. Antioxidant compounds with antihyperglycemic activities may be the best way to ameliorate the condition. The relation of antioxidant and glucose tolerance capacity of several compounds has been reported earlier [25]. The treatment with different doses of extract (100 and 200 mg/kg) significantly (*P* < 0.05) reduced blood glucose levels by 18.15%, 30.24% at 30min respectively, and significant (*P* < 0.05) reduction in the following hours was only observed at 200 mg/kg treated mice. Antihyperglycemic activity of the extract is comparable to the previous studies [10, 26]. The in vivo anti-diabetic activity of plant extracts has been correlated with their flavonoid and total phenolic content [27]. The presence of secondary metabolites such as alkaloids, polyphenols, flavonoid, glycosides, tannins and terpenoid in the ethanol extract of *P. sativum* may contribute to its antihyperglycemic activity and other medicinal values. Also, the extract contains significant amount of polyphenol and flavonoid content that is slightly higher in comparison to reported studies [10, 28, 29]. Different bioactive compounds of flavonoids, polyphenols, glycosides and terpenoids have been identified in the extract. β-sitosterol, identified in the extract has potential antidiabetic effect. It stimulates basal glucose uptake, the primary requisite for maintaining glucose homeostasis, through LKB1 (Liver Kinase B1) mediated AMP-activated protein kinase (AMPK) activation and also possesses insulin-like properties [30, 31]. On the other hands, polyphenolic ellagic acid potentiates pancreatic secretion, increases glucose uptake, liver and muscle glycogen content, decreases glucose intolerance and reduces glucose absorption by inhibiting glucosidase enzyme [32, 33]. The flavonoid, kaempferol improves insulin-stimulated glucose uptake in mature adipocytes [34] and naringenin suppresses carbohydrate absorption from intestine, exerts extra-pancreatic action [35]. One of the isolated compounds, β-amyrin improves glycemia possibly by its interaction with cannabinoid system [36]. These may be the possible mechanisms underlying the anti-diabetic activity of the extract.

The antioxidant potential of plant extract depends on the chosen method, concentration and physicochemical properties of components present in the extract. Vegetable as dietary sources contains significant amount of polyphenols that are responsible for multiple biological responses including antioxidant activity [37, 38]. In the present investigation extract showed dose dependent DPPH radical scavenging activity with the IC_50_ value of 158.52 μg/mL. Also in reducing power assay the extract showed moderate in action as free radical chain breaking agent. These results are comparable to that of previous studies [10, 28, 29]. Antioxidant activity of polyphenol identified in the extract namely ellagic acid, p-coumaric acid, naringenin, kaempferol has been reported [39, 40]. Also β-sitosterol has potential antioxidant effect [41]. These compounds act as hydrogen atom donor and neutralize DPPH• to DPPH-H and also break free radical induced chain reactions. All these identified compounds in the extract may be responsible for its antioxidant activity.

A number of studies have been reported the correlation of free radicals, antioxidants and protection of cellular essential components of insulin secretion and glucose uptake mechanism. Antioxidants may regulate insulin secretion, glucose uptake and consequent antihyperglycemic activity by several pathways namely up regulation of hepatic and adipocyte PPAR and GLUT4; phosphorylation of AMPK; activation of PPAR targetgenes; modulation of SREBP-1c; enhancement of anti-apoptotic AKT (also known as protein kinase B (PKB)) and Bcl-2 proteins, enhanced cAMP signaling and phosphatidylinositol-4,5-bisphosphate 3-kinase (PI3K).

## Conclusion

In the present study, we have evaluated the anti-hyperglycemic and antioxidant activities of the ethanol extract of *P. sativum*. The extract showed potentanti-hyperglycemic effect in oral glucose tolerance test in mice. The extract also demonstrated antioxidant effect. The presence of biologically active compounds ellagic acid and β-sitosterol could justify the results obtained. Our study also correlated to the chemical profile and traditional uses of the experimental edible herb that supported the traditional use of *P. sativum* as a promising natural pharmaceutical for combating diabetes.

## Abbreviations

AMPK: AMP-activated protein kinase
BHT: Butylated Hydroxy Toluene
DPPH: 2,2-diphenyl-1-picryldydrazyl
GAE: Gallic Acid Equivalent
HPLC: High Performance Liquid Chromatography
IC_50_: 50% Inhibitory Concentration
LCMS: Liquid Chromatography Mass Spectroscopy
LD_50_: Lethal dose in 50% test animal
LKB1: Liver Kinase B1
QE: Quercetin Equivalent
*R*^*2*^: Coefficient of Determination
ROS: Reactive Oxygen Species
RSD: Relative Standard Deviation
